# Multimodal imaging evaluation and management of orbital metastasis: experience at a single institution

**DOI:** 10.1007/s10792-026-03992-1

**Published:** 2026-03-03

**Authors:** Bernadete Ayres, Tassapol Singalavanija, Hakan Demirci

**Affiliations:** Department of Ophthalmology and Visual Sciences, W. K. Kellogg Eye Center, University of Michigan, 1000 Wall St, Ann Arbor, MI 48105 USA

**Keywords:** Cancer, Metastasis, Orbit, Imaging, Survival time

## Abstract

**Background and purpose:**

The orbit is an unusual site for metastatic cancer. This study evaluated the ultrasonographic and MRI/CT imaging features of orbital metastasis (OM) as well as demographic, clinical, radiological, management, and outcome.

**Methods:**

Retrospective, non-comparative single-institutional chart review of patients with OM. Records were evaluated for age at presentation, race, gender, laterality, site of primary tumor, imaging findings of orbital metastasis, treatment, and outcome.

**Results:**

There were 12 males and 15 females whose mean age at presentation was 60 years. Twenty-three patients (85%) had known primary cancer. Primary malignancies were breast carcinoma in 12 patients (44.5%), melanoma in 5 (18.5%), and lung carcinoma in 3 (11.1%). The most present findings were ocular motility disturbances (63%), proptosis (55%), and vision loss (19%). The lesions were well-outlined in 74%, located posterior to the equator in 59%, involved only one quadrant in 56%, and involved the extraocular muscles in 74%. Ultrasound was able to detect orbital or muscle lesions in 24 patients (89%). The shape, configuration, and location of the lesion and detection of extraocular muscle involvement agreed between MRI/CT and US exams. Treatment protocols included radiotherapy, chemotherapy, immunotherapy, hormone therapy and surgical excision. Seventeen patients (63%) had died of metastasis, with a mean overall survival time of 19 months after OM diagnosis.

**Conclusions:**

Breast carcinoma, melanoma, and lung carcinoma are the most common primary malignancies that metastasize to the orbit. OM tends to infiltrate the extraocular muscles. Ultrasound provides reliable parameters and can be used as a primary screening when evaluating suspected OM lesions. The survival time of patients with OM is generally poor.

## Introduction

Metastasis to the orbit is uncommon, with a reported frequency of 1–13% of all space-occupying lesions of the orbit [1–3]. Given its rarity, metastasis to the orbit may pose a diagnostic challenge, especially when the primary tumor is undiagnosed at the time of presentation, resulting in misinterpretation of metastasis as a primary orbital tumor. With continued improvements in treatment and survival for cancer patients, the frequency of detection of metastasis to unusual sites is increasing, and ophthalmologists should be aware of the significance of the clinical characteristics, imaging features and therapeutic options.

In this study, we evaluated the ultrasonographic and MRI/CT imaging features of orbital metastasis as well as demographic, clinical, radiological, management, and outcome analysis of 27 consecutive patients with orbital metastasis (OM) who were referred to an ocular oncology single center over a 10-year period.

## Material and methods

This study was conducted in accordance with the Declaration of Helsinki. The research protocol was approved by the Institutional Review Board at the University of Michigan.

A non-comparative retrospective review of the medical records was performed for patients with OM presenting at the Oncology Center at the Kellogg Eye Center, University of Michigan, between 2013 and 2023.

Demographic data collected included the patient’s age, gender, history of known cancer, site of primary cancer, presenting symptoms, and time interval between diagnosis of primary cancer and OM. The patients underwent a complete ophthalmological examination. The multimodal imaging protocol included MRI, CT scan, and ultrasonography. Radiographic characteristics collected included laterality, location (intraconal and/or extraconal, primary orbital quadrant, and axial region delimited by the globe equator in anterior to the equator and/or posterior to the equator), extension, shape, borders, and tissues involved. Diagnostic procedures, treatments (surgery, radiotherapy, chemotherapy, hormonal therapy), and follow-up data were also recorded. Follow-up time was calculated from the time of diagnosis of OM to the last clinical contact with the patient or death.

Inclusion criteria were patients with a known diagnosis of a primary tumor prior to OM or those who were diagnosed with a primary tumor following ocular presentation. Patients with secondary extension from a tumor originating from adjacent structures, as well as cases of lymphocytic disease, were excluded. A total of 54 eyes belonging to 27 individuals were included in the study.

Excel 2021 (Microsoft Corp., Redmond, WA, USA) was used for data collection, and SPSS V.28 (IBM Corp., Armonk, NY, USA) was utilized for statistical analysis. To compare two groups, the Student’s t-test was used when the distributions were normal. Relationships between variables were analyzed using Pearson Correlation Analysis. Statistical significance level was accepted as *p* < 0.05 for all tests.

## Results

We identified 27 patients diagnosed with OM between 2013 and 2023. The demographics and clinical characteristics are shown in Table 1. The mean age of the patients was 60 years (range, 38–86), and 15 were female (56%) and 12 were male (44%).

**Table 1:** Demographics and clinic characteristics of 27 patients with orbital metastasis

*Age (years)*	
Mean	60
Range	38–86
*Gender*	
Male	12 (44%)
Female	15 (56%)
*Known cancer patient*	
Yes	23 (85%)
No	4 (15%)
*Time interval from primary (months)*	
Mean	49
Range	2–264
*Primary tumor site*	
Breast carcinoma	12 (44.5%)
Cutaneous melanoma	3 (11.1%)
Lung carcinoma	3 (11.1%)
Choroidal melanoma	2 (7.4%)
Other	7 (25.9%)
*Symptoms/signs*	
Ocular motility	17 (63%)
Proptosis	15 (55%)
Vision loss	5 (19%)
Globe displacement	4 (15%)
Palpable mass	2 (7%)

Most of the OM presented in patients with known primary tumors (85%). The mean time lag between diagnosis of the primary tumor and orbital presentation was 49 months (range, 2–264).

Breast carcinoma was the most common primary tumor (44.5%), followed by cutaneous melanoma (11.1%), lung carcinoma (11.1%), choroidal melanoma (7.4%), and others (25.9%), including renal carcinoma, prostatic adenocarcinoma, uterine adenosarcoma, bladder carcinoma, and laryngeal neuroendocrine carcinoma.

The most common presenting symptoms and signs were limitation of the ocular motility in 17 patients (63%), proptosis in 15 patients (55%), vision loss secondary to optic neuropathy or radiation retinopathy in 5 patients (19%), globe displacement in 4 patients (15%), and palpable mass in 2 patients (7%).

Twenty-four patients (89%) had unilateral involvement, and 3 patients (11%) had bilateral involvement; 2 with breast lobular adenocarcinoma and 1 with cutaneous melanoma. There was no predominance of one eye in the unilateral group; the right orbit was involved in 12 patients (44.5%), and the left orbit was involved in 12 patients (44.5%) (Table 2).

**Table 2:** Multimodal imaging features of a case series of orbital metastasis

*Laterality*	
Right	12 (44.5%)
Left	12 (44.5%)
Bilateral	3 (11%)
*Tissue infiltrated*	
Muscle	20 (74%)
Fat	14 (63%)
Bone	9 (33%)
Lacrimal gland	5 (18%)
Optic nerve	3 (11%)
*Configuration of lesion*	
Irregular shape	11 (41%)
Fusiform	11 (41%)
Oval	4 (15%)
*Quadrant location*	
Inferior	15 (56%)
Temporal	15 (56%)
Superior	10 (37%)
Nasal	11 (41%)

**Fig. 1:**
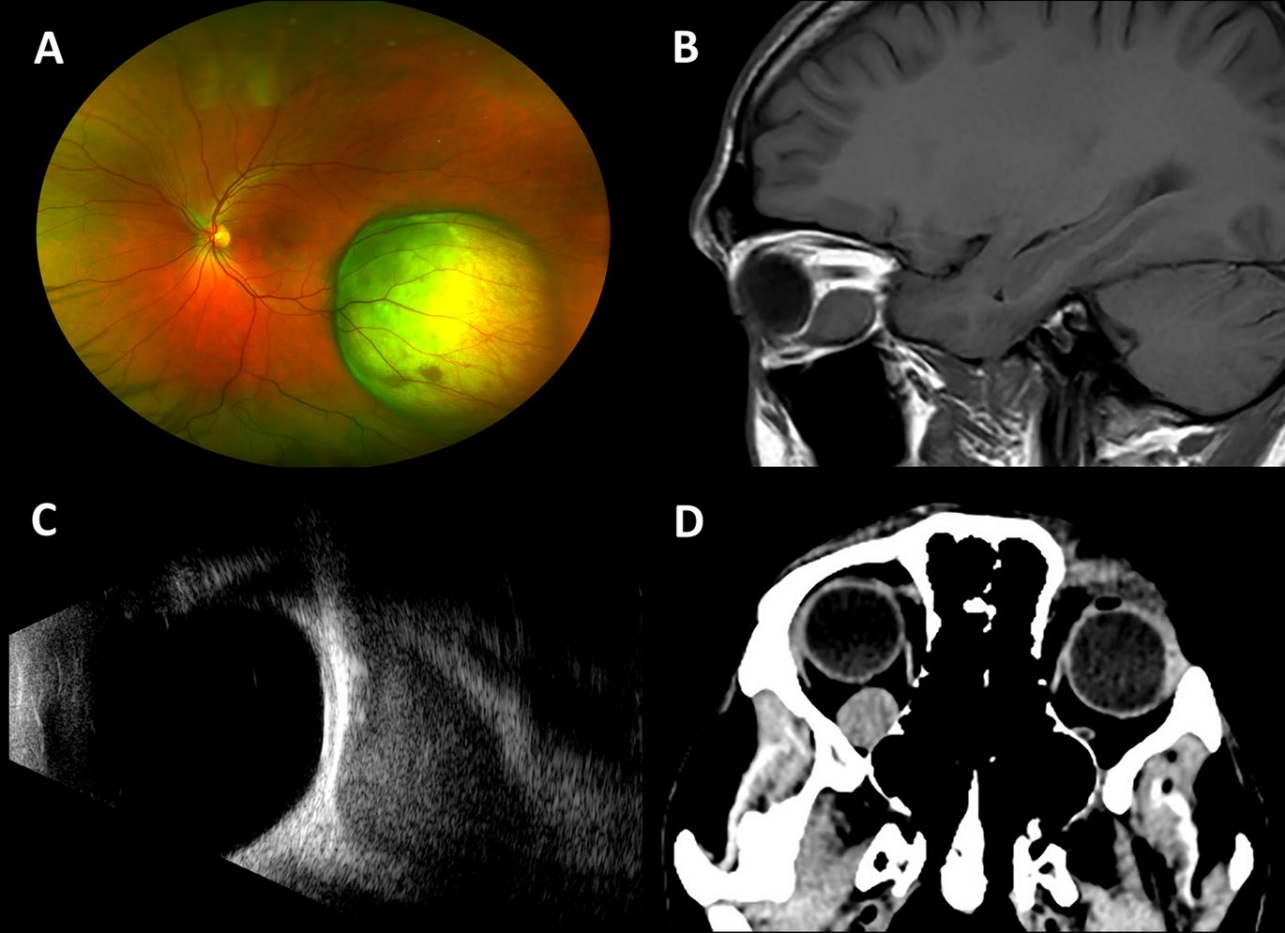
Multimodality imaging in a patient with orbital metastasis. **A** Fundus photography shows a choroidal melanoma in the left eye six years before the diagnosis of orbital metastasis. **B** MRI in the sagittal plane shows an enlargement of the inferior rectus muscle. **C** Ultrasound B-scan in longitudinal approach shows a low reflective enlargement of an extraocular muscle. **D** CT scan in the axial plane shows a well-outlined, right-sided orbital lesion, in the intraconal space

On review of imaging, 17 patients (63%) had US, MRI and CT scan exams, 9 patients (33%) had US and MRI, and 1 patient (4%) had only US and CT scan. There were 16 extraconal lesions (59%), 8 both intra and extraconal (30%), and 3 intraconal lesions (11%). Regarding the number of affected quadrants, most cases had only one quadrant involved (15 patients, 56%), whereas multiple quadrants involvement was less frequent (12 patients, 44%). The inferior quadrants were the most frequently involved, with 56%, followed by the temporal, 56%, superior with 37%, and nasal, 41%. The lesion was located posteriorly to the equator in 16 patients (59%), anterior and posterior to the equator in 8 patients (30%) and located in the anterior orbit in 3 patients (11%) (Fig. 1). MRI/CT and US detected the orbital lesions and identified their shape, configuration, and location with 89% agreement.

The lesions affected the extraocular muscles in 20 patients (74%), the orbital fat in 17 patients (63%), the, the bony orbit involvement, either remodeling or erosion, was detected in 9 patients (33%), lacrimal gland in 5 patients (18%), and the optic nerve was involved in 3 patients (11%).

Extraocular muscle involvement was in a single muscle in 12 patients (44%) and multiple muscles in 8 patients (30%). The most affected extraocular muscles were the inferior rectus in 9 orbits (33%), followed by the superior rectus in 8 orbits (30%), the lateral rectus in 8 patients (30%) and then the medial rectus in 7 patients (26%). The muscle tendon was spared in 50% of the cases. When comparing muscle lesions identified with US and MRI exams, there was 88% agreement in both exams independently performed (rs (27) = 0.67, *p* = 0.0002). In addition, the extraocular muscles were involved in all cases of breast and skin cancers but not in cases of lung carcinomas, blader prostate and uterus cancers. In patients with breast cancer, there was no predominance in a specific rectus muscle. In addition, the obliques or lid levator were not involved. However, the group of patients with melanoma tends to impact the inferior rectus; once again, no involvement of the obliques or lid levator was detected.

Concerning configuration and margin, the tumors were well-outlined in 20 patients (74%) and poor-outlined in 7 patients (26%). The shape was irregular in 11 tumors (41%), fusiform in 11 tumors (41%), and oval in 4 tumors (15%). The fusiform shape was found most in the cases of extraocular muscle involvement.

All patients underwent an ultrasonographic imaging exam. The sonographic characteristics are described in Table 3. Tumor masses were detected in 24 patients (89%). In 3 patients (11%), the lesion could not be detected with ultrasound due to their deep location at the orbital apex. Nineteen tumors (79%) were homogeneous, and 5 tumors (21%) had heterogeneous structures. The reflectivity was low in 18 tumors (75%), medium in 5 tumors (21%), and medium–high in only one case (4%). OM from breast carcinomas were predominantly homogeneous in structure (92%) and showed reflectivity varying from low to medium (100%). OM from melanomas had homogeneous structures (80%) and low to medium reflectivity (100%). Bony orbit was normal in 18 patients (67%), and bony defect was detected in 9 patients (33%), 6 had breast cancer, 1 had blader cancer, 1 had prostate cancer and 1 had melanoma. There was 78% agreement between US and CT scans in detecting bony defects (rs (16) = 0.882, *p* < 0.0001). On ultrasound B-scan, bone erosion and bone remodeling can be visualized as a surface defect of the hyperreflective bone line.

**Table 3:** Sonographic characteristics of orbital metastasis lesions

*Structure*	
Homogeneous	79%
Heterogeneous	21%
*Reflectivity*	
Low	75%
Medium	21%
Medium–high	4%
*Bony involvement*	
No involvement	67%
Bony erosion/invasion	33%

Fifteen patients (55%) underwent positron emission tomography and multiple metastatic sites were found in all patients.

OM was diagnosed based on clinical and imaging characteristics in 17 patients (63%), and 10 patients (37%) had orbital biopsy to confirm the diagnosis.

Various treatment protocols were used based on the orbital and systemic involvement and systemic prognosis of the patient. Six patients (22.3%) were treated with a combination of chemotherapy and radiotherapy, 4 patients (14.8%) with chemotherapy, radiotherapy, and hormonal therapy, 4 patients (14.8%) with immunotherapy therapy, 3 patients (11.1%) with hormonal therapy, 3 patients (11.1%) with chemotherapy, radiotherapy and immunotherapy, 2 patients (7.4%) with radiotherapy and immunotherapy, 2 patients (7.4%) with surgical debulking and immunotherapy, 1 patient (3.7%) with chemotherapy, 1 patient (3.7%) with radiotherapy and 1 patient (3.7%) with chemotherapy and immunotherapy. The mean external beam radiotherapy dose was 30 Gy. The radiation dose was determined by the treating radiation oncologist based on the primary malignancy. Among the 15 patients who received radiotherapy, the most common total dose was 30 Gy (8 patients), followed by 20 Gy (4 patients); one patient each received total doses of 24 Gy, 45 Gy, and 60 Gy. Systemic chemotherapy regimens and duration, as well as the decision to incorporate immunotherapy, were determined by the treating medical oncologist.

At last contact, 17 patients (63%) had died after a mean follow-up of 19 months from diagnosis of OM to death (range, 3–61). Seven patients (26%) are alive after a mean follow-up of 22 months since OM diagnosis (range 3–62). Three patients (11%) were lost to follow-up. The primary tumors that had less than 1 year of follow-up between the diagnosis of orbital metastasis and death were choroidal melanoma in 2, skin melanoma in 1, prostate cancer in 1, and kidney cancer in 1 patient. We did not observe higher mortality in patients with more aggressive orbital involvement features, such as bone erosion.

## Discussion

Orbital metastasis is usually observed in adults. In our series, OM occurred in patients with a mean age of 60 years. We found that most metastasis occurred unilaterally, and there was no preference of laterality in the involvement of the eye.

Similar to previous reports, breast carcinoma was the most frequent primary cancer to metastasize to the orbit, accounting for 44.5% of our patients. This finding correlates to the slight predominance of female patients observed in our series. The second most frequent primary cancer varies in different previous studies. El-Hadad et al. and Valenzuela et al. reported that melanoma was the second most common tumor to metastasize to the orbit [4, 5]. Shields et al. reported that prostate carcinoma was the second leading cause of OM [2]. Hepatocellular carcinoma was the most common primary tumor metastasizing to the orbit in Egyptian patients [6]. These differences may be related to the referral pattern of the institution or geographic area of a certain study. In this study, melanomas, either cutaneous or choroidal, were the second most frequently encountered primary cancer. In our series, 85% of the patients had a known primary tumor, and the mean time interval between diagnosis of the primary tumor and orbital presentation was 49 months. These results are in line with previous studies, which report an incidence of 67–95% of patients with a known history of malignancy and a mean time 12–71 months between the initial diagnosis and the onset of symptoms of OM. [2, 4–8]

Orbital metastasis frequently manifests clinically with an abrupt onset of rapidly progressive symptoms [5]. Compared with previous similar studies, our patients often presented with disturbances of ocular motility, proptosis, vision loss, and globe displacement. Less frequent and usually associated with scirrhous breast carcinoma, enophthalmos, which can be caused by the fibrosis of retrobulbar stromal tissue, can be the presenting sign of OM [2, 4, 7, 9, 10]. These signs and symptoms may be progressive over weeks to few months.

Multimodality imaging is critical in characterizing orbital tumors. Using US, MRI, and CT scan, our study showed well-outlined tumors, with irregular or fusiform shapes, occupying the extraconal and post-equatorial spaces, and equally distributed in the orbital quadrants in most cases. There was 89% agreement between these 3 orbital imaging techniques in the detection of shape, location, and intrinsic imaging features of OM.

In our study, we observed a higher rate of extraocular muscle involvement (74%). This tendency of OM to infiltrate the muscles explains why ocular motility limitation was the most presenting sign in our series. The recti muscles were particularly affected in all patients without predominance of a specific rectus muscle while oblique muscle involvement was observed in only one patient. Several studies have noted the propensity for extraocular muscles from metastatic melanoma and breast carcinoma [5, 8, 10–13]. Similarly, we noted that the extraocular muscles were involved in all cases of breast and skin cancers but not in cases of lung carcinomas. There was 88% agreement in both ultrasound and MRI in detection of the extraocular muscle involvement independently performed (rs (27) = 0.67, *p* = 0.0002).

Orbital imaging using ultrasound as a screening method for detection of orbital tumors is an effective and readily-available technique [14–17]. In our study, the ultrasound was able to detect OM in 89% of the cases. The lesions located deep in the orbital apex can not be detected with high-frequency ultrasound due its limited penetration. Most of lesions demonstrated well-outlined masses with a homogeneous structure and low reflectivity. Furthermore, enlargement of the extraocular muscles can be assessed with ultrasound. Bone erosion or remodeling was found in 33% of the OM. MRI and CT scan are still the principal methods of evaluating OM. The main disadvantage of ultrasonography is the lack of deep penetration around the orbital apex. Because OM involves the orbital soft tissues in most cases MRI provides better resolution for assessing the orbit for suspected space occupying masses. CT scan is more useful in patients with prostate cancers, which commonly infiltrate the orbital bony, inducing osteoblastic or osteoclastic reactions. Positron emission tomography (PET) with (18F)-fluorodeoxyglucose (FDG) is a well-established tool for staging these patients. PET/CT combines the functional imaging of PET scans with the anatomical imaging of CT scans. In the past years, combined PET/CT imaging has emerged as a diagnostic modality, and its importance has been proven in diagnosing and monitoring patients with metastasis [4, 18]. An incisional biopsy or a fine-needle biopsy aspiration may be necessary to confirm the clinical suspicious of an OM.

The treatment of the OM aims to control the growth of the metastasis, preserve the vision function, and maximize the patient’s quality of life. The selection of a systemic therapy, a local therapy, or a combination of these is based on several factors, such as age, life expectancy, comorbidities, pathologic findings, and biological behavior of the tumor. Radiotherapy is a well-established palliative therapeutic option, improving the clinical and functional status of patients with OM. In our current series, the main modality was radiotherapy as the sole in 1 patient (4%) or in combination in 15 patients (56%). Chemotherapy, immunotherapy, and hormonal therapies proved to be significantly effective in concurrently treating both the primary cancer and the OM. Immunotherapy as adjuvant to chemotherapy has been reported to improve survival in patients with metastatic melanoma [12]. Tumor debulking can prompt decompress with relief of masses effect and increases the effectiveness of complementary therapies.

The prognosis of the patient with OM is very poor. In our data, the overall survival of patient following their diagnosis of OM was 19 months. A study conducted by El-Hadad et al. showed a mean overall survival rate of 17 months [4]. Shields et al. reported the longest survival in patients with metastatic carcinoid tumors and breast carcinoma, with a mean of 60 and 22 months, respectively, in contrast with patients with lung cancer who had a least favorable prognosis with a mean survival of 4 months [2]. Recently, treatment with immunotherapy for patients with cutaneous melanoma metastatic to the orbit appears to contribute to a longer survival rate. [12]

## Conclusions

In summary, we describe our experience with 27 consecutive patients with orbital metastasis. Breast carcinoma, melanoma, and lung carcinoma were the most frequent primary cancers; however, various cancer types can spread to the orbit. A high incidence of extraocular muscles involvement was found in this series. Ultrasound is a readily available and useful imaging tool for the primary screening for orbital space-occupying lesions, providing accurate detection. MRI remains the gold standard for imaging OM. Multimodal imaging offers complementary advantages to provide a more complete and accurate diagnosis. Ultrasonography is a noninvasive, first-line imaging modality that enables accurate detection and tissue differentiation of orbital and periorbital lesions. Ophthalmic ultrasound is a valuable adjunct to MRI and CT scan in the evaluation of orbital lesions. PET/CT imaging can be employed for detecting cancer of unknown primary sites and stage disseminated disease. The survival time of patients with OM is poor. Prompt diagnosis is one of the most critical factors that can guide the management of the disease, improving the prognosis, preserving the vision, and improving the patient’s survival time.

## Data Availability

No datasets were generated or analysed during the current study.
